# An Important Change

**Published:** 1884-03

**Authors:** 


### An Important Change.

The change in the management of the Erie County Insane Asylum whereby Dr. Charles A. Ring has assumed the Superintendency of that institution is a movement of so much importance that we cannot forbear to notice it. With the increase in population of our city, the number of insane collected in the County Asylum has been largely augmented, and as a consequence the necessity of a Superintendent possessed of a thorough medical education and a familiarity with insanity in its scientific management has received deserved recognition. At last, this principle has been put in operation, and the selection of the Superintendent has been made from professional fitness rather than from political influence. We cordially endorse the appointment. Dr. Ring has occupied for several years the position of resident physician at the asylum and has demonstrated by long and faithful labor his ability and capacity for the position to which he has been appointed. The profession will thoroughly endorse not only the principle upon which the change in management is based, but also the personal and professional fitness of the new Superintendent. There is no reason why our County Asylum in which are aggregated large numbers of the insane should not be placed under stricter management. The State recognizes this principle in the superintendency of its institutions, and the benefits received therefrom are among the blessings which flow from these sources to the public at large. The county has needed for a long time just such a change. It comes late, but not so late as to prevent Dr. Ring from instituting necessary reforms and thus accomplishing much good to the unfortunate inmates over whom he has been placed. We shall look therefore for great improvement in the asylum that will make it compare in its results with the State Asylums, which have long been a pride of our Commonwealth.

